# Adverse COVID-19 experiences and health-related quality of life in cancer survivors: indirect effects of COVID-19-related depression and financial burden

**DOI:** 10.1186/s41687-023-00601-y

**Published:** 2023-07-17

**Authors:** Laura M. Perry, John D. Peipert, Sheetal M. Kircher, Jackelyn Cantoral, Frank J. Penedo, Sofia F. Garcia

**Affiliations:** 1 Northwestern University Feinberg School of Medicine, Chicago, IL USA; 2 Sylvester Comprehensive Cancer Center, University of Miami, Miami, FL USA

**Keywords:** Cancer, COVID-19, Health-related quality of life, Patient-reported outcomes, Depression, Financial burden

## Abstract

**Background:**

Cancer survivors are at greater risk for poor health outcomes due to COVID-19. However, the pandemic's impact on patients’ health-related quality of life (HRQoL) is not well known. This study hypothesized that cancer survivors' adverse COVID-19 experiences would be associated with worse HRQoL. Further, this association would be moderated by psychosocial resiliency factors (perceived social support, benefits, and ability to manage stress) and mediated by psychosocial risk factors (anxiety, depression; health, financial and social concerns).

**Methods:**

1,043 cancer survivors receiving care at Northwestern Medicine completed a cross-sectional survey on COVID-19 practical and psychosocial concerns from 6/2021 to 3/2022. Participants reported on 21 adverse COVID-19 experiences (e.g., COVID-19 hospitalization, death of family/friends, loss of income, medical delays). The survey assessed 9 psychosocial factors related to COVID-19: anxiety, depression; health care, financial, and social disruptions; health care satisfaction; social support, perceived benefits, and stress management skills. The FACT-G7 assessed HRQoL. Hypotheses were tested in a structural equation model. The number of reported adverse COVID-19 experiences was the primary (observed) independent variable. The dependent variable of HRQoL, and the proposed mediating and moderating factors, were entered as latent variables indicated by their respective survey items. Latent interaction terms between the independent variable and each resiliency factor tested moderation effects. Analyses were adjusted for demographic and COVID-specific variables.

**Results:**

Participants were, on average, aged 58 years and diagnosed with cancer 4.9 years prior. They were majority female (73.3%), White (89.6%), non-Hispanic/Latino (94.5%), college-educated (81.7%), and vaccinated for COVID-19 (95.5%). An average of 3.8 adverse COVID-19 experiences were reported. Results of structural equation modeling demonstrated that the association between adverse COVID-19 experiences and HRQoL was explained by indirect effects through COVID-19-related depression (β = − 0.10*,* percentile bootstrap 95% CI − 0.15 to − 0.07) and financial concerns (β = − 0.04, percentile bootstrap 95% CI − 0.07 to − 0.01). Hypotheses testing moderation by resiliency factors were not significant.

**Conclusions:**

Adverse COVID-19 experiences were associated with higher depression symptoms and financial concerns about COVID-19, and in turn, worse HRQoL. Oncology clinics should be cognizant of the experience of adverse COVID-19 events when allocating depression and financial support resources.

**Supplementary Information:**

The online version contains supplementary material available at 10.1186/s41687-023-00601-y.

## Abstract (plain language summary)

We conducted an online survey of cancer survivors receiving treatment at Northwestern Medicine in Chicago, Illinois. Participants responded to a list of 21 adverse experiences related to the pandemic, such as COVID-19 hospitalization, death of family/friends, loss of income, and medical delays. They also responded to questionnaires measuring their degree of anxiety, depression, daily disruptions, health disruptions, financial disruptions, social support, perceived benefits, and ability to manage stress during the pandemic. Lastly, they responded to a questionnaire on health-related quality of life, capturing their physical symptoms, emotional symptoms, and satisfaction with life. Our survey found that people who had a greater number of adverse COVID-19 experiences had higher levels of depression and financial burden, which in turn was associated with worse health-related quality of life.

**Supplementary Information:**

The online version contains supplementary material available at 10.1186/s41687-023-00601-y.

## Introduction

Since its onset in March 2020, the COVID-19 pandemic has had a widespread and lasting impact on people's lives. As of December 2022, it has resulted in nearly 100 million infections and over 1 million deaths in the United States alone [1]. Individuals infected with COVID-19 experience acute illness ranging from mild to life-threatening, and 15–30% will develop chronic long COVID symptoms such as fatigue, brain fog, memory difficulties, difficulty breathing, and other symptoms that can interfere with daily life and well-being [2, 3]. In addition to the experience and threat of physical illness, the pandemic has resulted in other stressors including social isolation, work disruptions, loss of income, healthcare disruptions, caregiving burden, and bereavement. As a result, the pandemic is estimated to cost approximately $16 trillion in lost income and productivity due to lockdowns, premature death, long-term health impairment, and mental health impairment [4, 5]. Individuals with a history of cancer are at an especially high risk of experiencing poor outcomes during the pandemic, due to the compounding physical, emotional, and financial demands of both the pandemic and their illness [6–8].

A large body of literature has been devoted to addressing the multifaceted issue of HRQoL in oncology, but little research has focused on this topic during the COVID-19 pandemic [6, 7, 9]. HRQoL refers to the impact of health issues on a person's life across physical, emotional, and social domains [10]. Past research has demonstrated that psychosocial factors can serve as both risk factors and protective factors against poor HRQoL in cancer survivors. For instance, increased social support, benefit-finding, and perceived coping ability are associated with better HRQoL [11, 12]. In contrast, increased emotional distress, such as anxiety and depressive symptoms, and financial burden predict worse HRQoL [11]. The pandemic has exacerbated these risk factors, due in part to fear and uncertainty about health status, loneliness, healthcare disruptions, and threats against job security and finances [6–9]. However, many cancer survivors may exhibit resilience that can mitigate the pandemic's impact on HRQoL [13, 14]. More research is needed to capture a comprehensive assessment of psychosocial factors to evaluate which are most important for explaining risk for and protection against decreased HRQoL in cancer survivors during the pandemic.

To help fill this gap, we conducted a comprehensive survey of psychosocial risk and resiliency factors for oncology settings during the COVID-19 pandemic. The present study had the following hypotheses: (1) more COVID-19 adverse experiences will be associated with decreased HRQoL, (2) resiliency factors of social support, perceived benefits, and ability to manage stress will moderate the relationship between COVID-19 adverse experiences and HRQoL, where participants with greater resiliency will have a significantly weaker association among these measures, (3a) more COVID-19 adverse experiences will be associated with COVID-19 specific psychological distress and disruptions in health care, finances and social relations, 3b) COVID-19 distress and disruptions will in turn be associated with decreased HRQoL, and 3c) the association between COVID-19 adverse experiences and HRQoL will be mediated by COVID-19 distress and disruptions.

## Method

### Participants and procedures

From 6/2021 to 3/2022, cancer survivors at Northwestern Medicine cancer centers that met study inclusion and exclusion criteria were recruited to participate in a brief cross-sectional online survey. Inclusion criteria included (1) visit within the last year at Northwestern Medicine cancer clinics, (2) ICD-10 confirmed cancer diagnosis, (3) active email address, (4) able to read English, and (5) age 18 or older at time of cancer diagnosis. Exclusion criteria included (1) living outside of the USA, and (2) declined to be contacted for research. Eligible participants were identified by accessing data in Northwestern Medicine's Enterprise Data Warehouse (EDW) and then sent an invitation describing the study via email or the electronic health record patient portal. The EDW serves as a repository of all Northwestern Memorial electronic health records and contains information on the inclusion and exclusion criteria. Patients were able to indicate interest within the invitation message, and those who did were sent an email from REDCap [15] with the survey link. If a patient did not respond to the initial study invitation, up to two reminders were sent via the patient portal, REDCap, phone call, or text message. Patients were given the option to opt-out of any subsequent emails, phone calls, or texts as part of the e-consent process. All procedures were approved by the Northwestern University Institutional Review Board (STU00213846)).

### Sample size determination

Sample size was determined a priori based on achieving sufficient power to detect hypothesized indirect effects. To calculate power, we constructed a Monte Carlo simulation of a likely latent mediation model in the Mplus software. Our model had 3 latent factors [factor 1–3 (F1–F3)] with 3 observed indicators each. We specified direct effects from F1 to F2 and F3, and from F2 to F3, implying an indirect effect from F1 to F3 as well. Therefore, we were also able to test power to detect both direct and indirect effects. We made conservative assumptions with relatively low magnitude regression betas (Β = 0.25) for the direct effects and indirect effect (= 0.16), low factor loadings (= 0.40), and a sample size of 1000 patients. We specified 10,000 replications for the simulation. The power to detect the direct and indirect effects was determined by examining the number of replications that found significant effects for these paths. Under this scenario, our simulation found 91% power to detect the direct effect and 80% power to detect the indirect effect. Although this was not the final model we ended up testing (see Statistical Analysis), a sample size of at least 1000 is still sufficient for the size of our tested model and anticipated effect sizes based on guidelines for SEM power analyses [16, 17].

### Measures

The survey assessed participants’ experiences thus far during the COVID-19 pandemic (e.g., exposure, risk factors, testing, isolation, seropositivity, hospitalization, loss of family or friends); COVID-19 specific psychological distress (e.g., fear, anxiety and depressive symptoms); health, financial and social disruptions; perceived benefits and social support; and HRQoL.

### Demographic characteristics

Participants responded to questions on their demographics: age, gender, race, ethnicity, relationship status, education, employment status, income, and insurance status. They also reported on clinical variables including date of cancer diagnosis and COVID-19 vaccination status.

### Adverse COVID-19 experiences

Adverse COVID-19 experiences were assessed using a recently developed investigator-designed list from a previous study examining the impact of COVID-19 on cancer survivors [18]. Similar to other studies evaluating COVID-related stressors [19, 20], participants indicated whether or not they had experienced adverse COVID-19 experiences [18]. Specifically, they reported on the following risk factors or associated symptoms during the COVID-19 pandemic: (1) age 60 years or older, (2) high-risk comorbidities (e.g., diabetes, hypertension, kidney disease, respiratory disease), (3) international travel or travel to COVID-19 hotspots, (4) exposure to someone who tested positive for COVID-19, (5) visiting/working in a nursing home or hospital, (6) fever, (7) dry cough, and (8) shortness of breath. Participants also indicated whether they had any other adverse experiences from the following list: (9) tested positive for COVID-19, (10) currently experiencing COVID-19 symptoms, (11) hospitalized for COVID-19, (12) family member or member of household tested positive for COVID-19, (13) family member or member of household died of COVID-19, (14) friend, co-worker, or neighbor diagnosed with COVID-19, (15) friend, co-worker, or neighbor died of COVID-19, (16) lost job or primary source of income due to COVID-19, (17) spouse or partner lost job or primary source of income, (18) income decreased due to COVID-19, (19) delayed general medical appointment due to COVID-19, (20) delayed cancer care appointment or treatment due to COVID-19, 21) delayed emergency room or urgent care visit due to COVID-19. Responses to the 21 indicators were coded as 1 = yes or 0 = no, and then summed to create an index variable representing the total number of adverse COVID-19 experiences for each participant.

### Practical and psychosocial experiences questionnaire (COVID-PPE)

The proposed mediators and moderators in our analysis were captured by a recently developed and validated questionnaire assessing COVID-19-related practical and psychosocial concerns [18]. The questionnaire includes subscales measuring COVID-19-related depression, anxiety, health disruptions, daily disruptions, satisfaction with healthcare provider response to the pandemic, financial burden, perceived benefits, social support, and stress management ability. The subscale measuring satisfaction with provider response to the pandemic was not included in analyses because it was not relevant to the present study hypotheses. Each subscale was assessed by 2 to 6 Likert-type scale items where participants are asked to rate statements from 0 (strongly disagree) to 4 (strongly agree). Subscales were represented in analyses as latent (unobserved) variables in a structural equation model. The questionnaire was developed and psychometrically evaluated with > 10,000 cancer survivors from two large metropolitan areas to confirm its underlying factor structure and internal consistency of subscales (αs = 0.73–0.90).

### Health-related quality of life (HRQoL)

The survey also included the Functional Assessment of Cancer Therapy-7 (FACT-G7) which is a well-validated and commonly used measure of HRQoL in oncology [21] and the primary dependent variable of this study. The scale includes five items capturing common symptoms of cancer (fatigue, nausea, sleep, pain, illness anxiety) and two items assessing overall satisfaction with life and quality of life, rated from 0 (not at all) to 4 (very much). After reverse-scoring four of the items, a latent (unobserved) variable was specified in the SEM model, with higher scores indicating better HRQoL.

### Statistical analysis

All analyses were conducted in R version 4.2.1. Before undertaking analyses, data were screened for eligibility and missing values. Next, descriptive statistics were used to characterize the sample's demographic and clinical characteristics, as well as the distribution of adverse COVID-19 experiences. Last, hypotheses were tested simultaneously in a structural equation path model using the Lavaan package in R [22].

### Confirmatory factor analysis

**Fig. 1:**
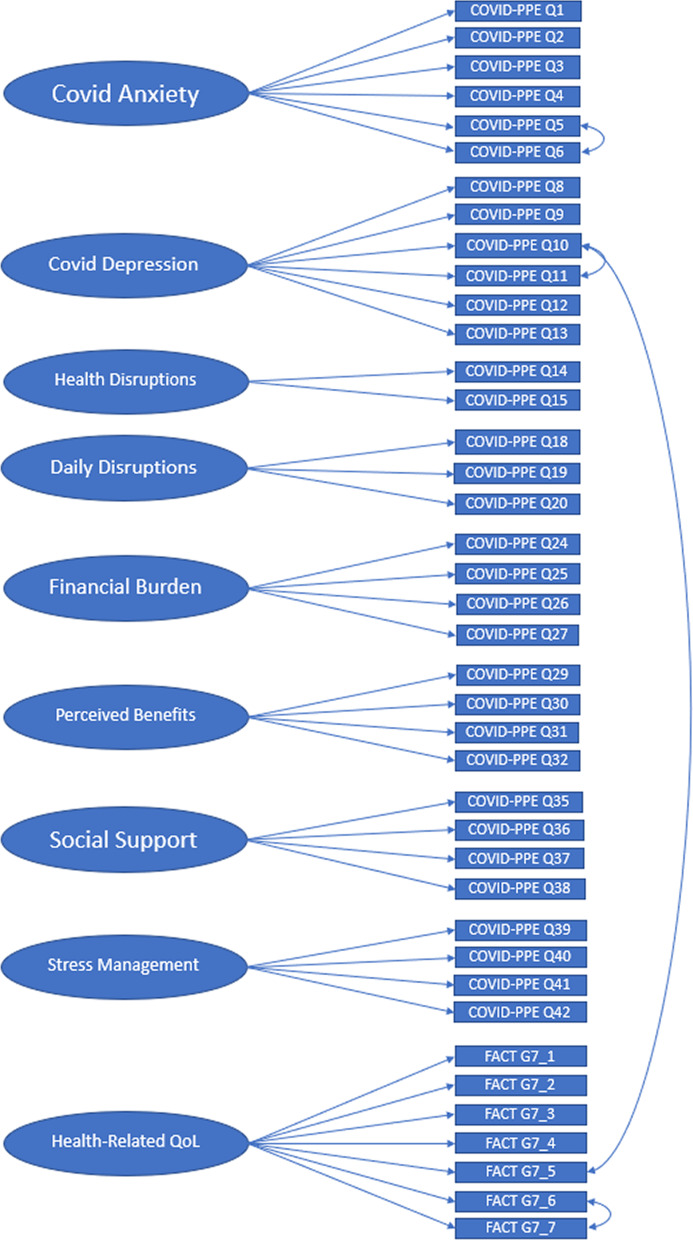
Hypothesized Measurement Model. *Note.* CFI = 0.90. TLI = 0.89. RMSEA = 0.05. SRMR = 0.06. For simplicity, arrows depicting variances and covariances between latent variables are not shown

Our hypothesized moderating, mediating, and outcome variables were represented in the model as latent variables indicated by their respective scale items. Before testing our hypotheses in a path model, we conducted a confirmatory factor analysis to verify our underlying measurement model (Fig. 1). Latent variables were allowed to covary with one another, and their variances were fixed to 1 to identify the model. Residuals between a handful of items with overlapping content were allowed to covary after examining modification indices to improve fit. To account for missing data, Full Information Maximum Likelihood (FIML) estimation was used for the analysis. We defined the following values as indicative of acceptable model fit: CFI and TLI ≥ 0.90, RMSEA ≤ 0.08, and SRMR values ≤ 0.10 [23].

### Structural equation modeling

**Fig. 2:**
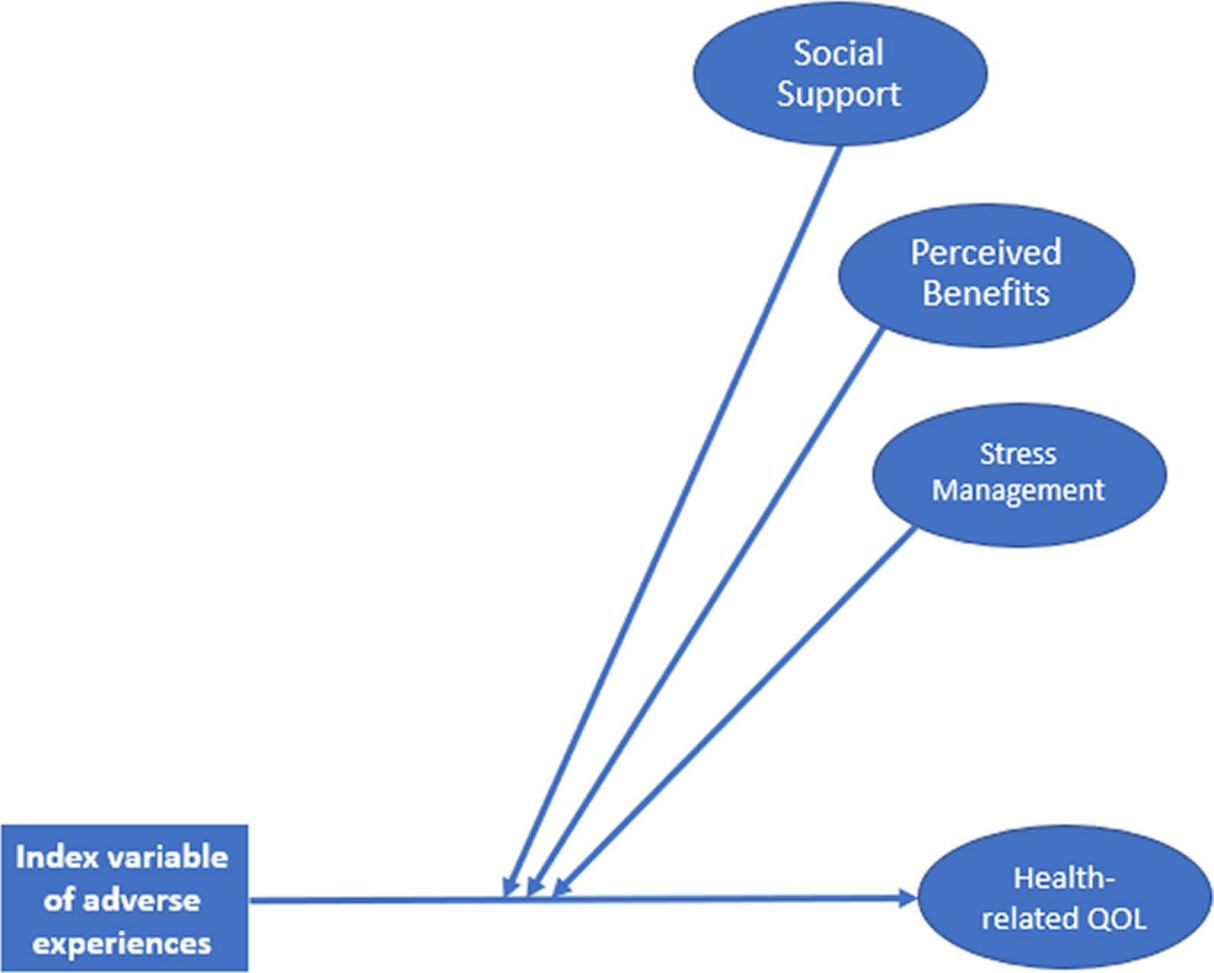
Hypothesized Moderators (Step 1). *Note*. CFI = 0.87. TLI = 0.86. RMSEA = 0.05. SRMR = 0.05. Latent moderators and HRQoL were measured identically to Fig. 1. The present model controlled for the following covariates: age, gender, race, ethnicity, time since diagnosis, COVID-19 vaccination status, and time since the vaccine was publicly available in the state of Illinois. Latent moderators were allowed to covary with one another. Arrows depicting covariate paths, latent variable indicators, variances, and covariances were left out of the figure for simplicity

The structural equation path model testing our hypotheses was built in two steps. In the first step, one regression model was specified (see Fig. 2), predicting our latent dependent variable (FACT-G7 score) from the observed independent variable (adverse COVID-19 index variable), our three latent moderating variables (social support, perceived benefits, stress management), and their interactions with the independent variable. The latent moderators and dependent variable were measured identically to the accepted CFA model. Interactions were captured with product-indicator latent interaction terms [24]. With this method, the product between the independent variable and each indicator item of the latent moderators was computed and then orthogonalized using double-mean centering in the semTools package within R [25, 26]. Next, three latent interaction variables were specified in the Lavaan model, each indicated by the product-indicator variables corresponding to a proposed moderator (social support, perceived benefits, stress management). The regression model was adjusted for observed covariates of age, gender, race, ethnicity, time since diagnosis, COVID-19 vaccination status, and time since the vaccine was publicly available in the state of Illinois. The Lavaan model was estimated using Full Information Maximum Likelihood (FIML) estimation to account for missing values on any of the independent or dependent variables. In the event of any significant interaction terms, a follow-up model would be specified to evaluate simple slopes of adverse COVID-19 experiences predicting HRQoL at low, average, and high levels of the moderator.

**Fig. 3:**
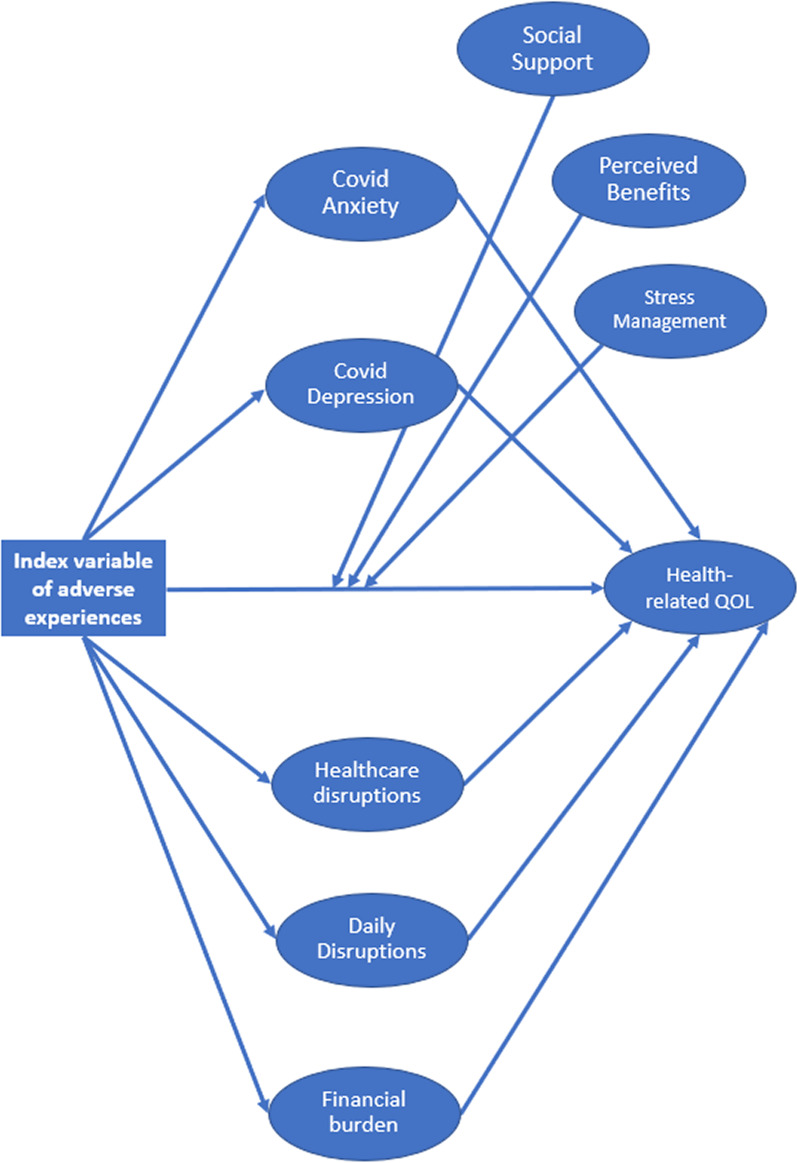
Hypothesized Moderators and Mediators (Step 2). *Note*. CFI = 0.87. TLI = 0.86. RMSEA = 0.04. SRMR = 0.05. Latent variables were indicated using the same measurement model from Fig. 1. All regression models controlled for covariates (See Table 3), and latent moderators and mediators were allowed to covary with one another. Arrows depicting covariate paths, latent variable indicators, variances, and covariances were left out of the figure for simplicity

In step 2, each of the five proposed mediators were added to the model, measured identically to the accepted CFA model. In this full model (Fig. 3), regression paths were added to test whether adverse COVID-19 experiences (observed independent variable) predicted the proposed latent mediating variables of COVID-19-related anxiety, depression, heath disruptions, daily disruptions, and financial burden. In addition, regression paths were added to test whether the five latent mediating variables predicted HRQoL (latent dependent variable). All regression models were adjusted for the observed covariates of age, gender, race, ethnicity, time since diagnosis, COVID-19 vaccination status, and time since the vaccine was publicly available in the state of Illinois. The model was estimated with Full Information Maximum Likelihood (FIML) to account for missing variables. Since the model estimated indirect effects, bootstrapped standard errors from a resampling size of 1,000 were used during model estimation. Indirect effects were evaluated for statistical significance using percentile bootstrap 95% confidence intervals.

## Results

### Data cleaning

A total of 2,419 patients accessed the REDCap survey link, with 1,101 consenting to proceed with the survey. Patients who were missing responses on all items of any multi-item scale were excluded from analyses (*n* = 57). One additional participant was removed due to indicating that they did not have a history of cancer. The final analytic sample was 1,043, and the degree of missingness ranged across items from 0 to 8.5%.

### Sample characteristics

Table 1 displays descriptive statistics for key sample characteristics. The sample consisted of mainly White (89.6%), non-Hispanic (94.5%), and female (73.3%) cancer survivors who were married or in a committed relationship (70.7%). On average, they were 58.1 years old (*SD* = 13.2) and diagnosed with cancer 4.9 years prior to participating (*SD* = 5.4). The sample was characterized by relatively high socioeconomic status, with 81.7% having a bachelor's degree or higher and 54.6% reporting their household income in the highest bracket of at least $100,000/year. Most participants were either employed full-time (44.3%) or retired (31.4%) and had either private insurance (64.9%) or Medicare (31.4%). The vast majority (95.5%) were vaccinated against COVID-19.

**Table 1:** Sample characteristics (*N* = 1,043)

Variable	N (%) or M (SD)
Age	58.1 (13.2)
Gender, female	765 (73.3%)
Hispanic/LatinX	57 (5.5%)
*Race*	
White	935 (89.6%)
Black	51 (4.9%)
Asian	33 (3.2%)
American Indian	7 (0.7%)
Other	23 (2.2%)
Married/In relationship	737 (70.7%)
Bachelor's degree or higher	852 (81.7%)
*Employment status*	
Full time employed	462 (44.3%)
Retired	328 (31.4%)
Part-time employed	94 (9.0%)
On disability	67 (6.4%)
Homemaker	35 (3.4%)
Unemployed	28 (2.7%)
Leave of absence	13 (1.2%)
Full-time student	4 (0.4%)
Unknown/missing	12 (1.2%)
*Insurance status*	
Private	677 (64.9%)
Medicare	334 (32.0%)
Medicaid	22 (2.1%)
Uninsured/Self-pay	5 (0.5%)
Don't Know/missing	5 (0.5%)
*Household income*	
Less than $15,000	14 (1.3%)
$15,000–$29,999	34 (3.3%)
$30,000–$59,999	110 (10.5%)
$60,000–$100,000	196 (18.8%)
More than $100,000	569 (54.6%)
Missing/Prefer not to answer	120 (11.5%)
Time since diagnosis, years	4.9 (5.4)
Vaccinated against COVID-19	996 (95.5%)
Covid-19 Adverse Experiences Index (sum of 21 experiences)	3.8 (2.2)

### Adverse COVID-19 experiences

**Fig. 4:**
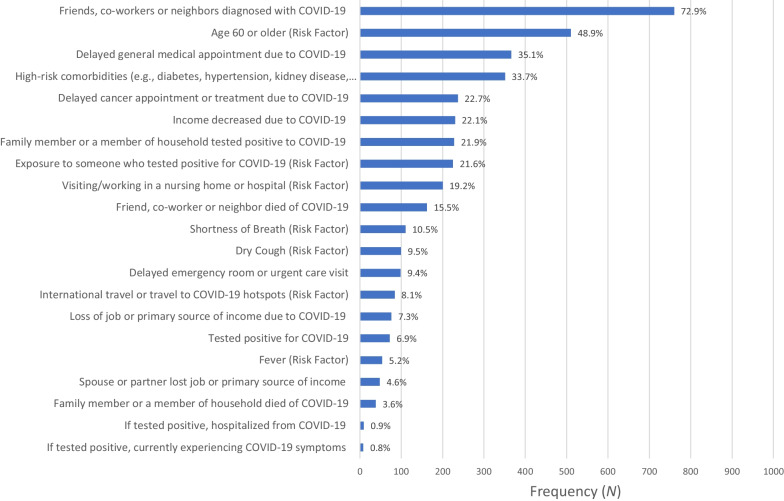
Frequency (*N*) and Percent (%) of Each Adverse COVID-19 Experience

Out of 21 possible adverse COVID-19 experiences, almost the entire sample (98.1%) reported at least one. On average, participants endorsed a mean of 3.8 adverse experiences (*SD* = 2.2; range = 0–16), with 33% of the sample reporting five or more. The five most common adverse experiences included (1) having a friend, coworker, or neighbor diagnosed with COVID-19 (72.9%); (2) being at risk due to age over 60 (48.9%,); (3) delaying a general medical appointment (35.1%); (4) being at risk due to comorbid conditions such as diabetes, hypertension, kidney disease, or respiratory disease (33.7%), and (5) delaying a cancer care appointment or treatment (22.7%). Figure 4 presents the frequency and percent for each assessed adverse experience.

### Confirmatory factor analysis

A confirmatory factor analysis testing our latent variable measurement model demonstrated an acceptable model fit (CFI = 0.90, TLI = 0.89, RMSEA = 0.05, SRMR = 0.06). As illustrated in Fig. 1, the latent variables were indicated by their respective scale items, and the residuals between four pairs of items were allowed to covary based on modification indices and overlapping item content. Specifically, we correlated the residuals between the items "I fear how the COVID-19 pandemic will impact my cancer care or recovery" (COVID-PPE item 5) and "I am concerned that cancer puts me at greater risk for being infected or dying from COVID-19" (COVID-PPE item 6); "I have experienced changes in my sleep" ( COVID-PPE item 10) and "I have experienced changes in my eating" (COVID-PPE item 11); "I have experienced changes in my sleep" (COVID-PPE item 10) and "I sleep very well" (FACT-G7 item 5); and "I am able to enjoy life" (FACT-G7 item 6) and "I am content with the quality of life right now" (FACT-G7 item 7). All items in the measurement model were strong indicators of their respective factors (factor loadings ≥ 0.40, see supplemental Table S1). Correlations between latent factors ranged in size from |*r|*= 0.01 to |*r|*= 0.71 (see Table 2).

**Table 2:** Latent factor correlations

	1	2	3	4	5	6	7	8	9
1. COVID Anxiety	1.00								
2. COVID Depression	0.57***	1.00							
3. COVID Health Disruption	0.26***	.45***	1.00						
4. COVID Daily Disruption	0.58***	0.71***	0.53***	1.00					
5. COVID Financial Burden	0.30***	0.47***	0.39***	0.38***	1.00				
6. Perceived Benefits	.12***	− .10**	− .05	− .01	.03	1.00			
7. Social Support	0.09*	− 0.07	− 0.11**	0.04	− 0.16***	0.48***	1.00		
8. Stress Management	− 0.08*	− 0.31***	− 0.20***	− 0.17***	− 0.13***	0.48***	0.62***	1.00	
9. Health-related QoL	− 0.35***	− 0.67***	− 0.36***	− 0.52***	− 0.45***	0.22***	0.29***	0.46***	1.00

### Structural equation modeling

#### Moderation hypotheses

After confirming our latent variable measurement model, regression paths were added to test our hypotheses. In Step 1, a regression model was specified to test whether the number of adverse COVID-19 experiences was associated with HRQoL, and whether this association was moderated by perceived social support, benefits, and stress management ability (CFI = 0.87. TLI = 0.86. RMSEA = 0.05. SRMR = 0.05). As hypothesized, results demonstrated that participants who reported a greater number of adverse COVID-19 experiences had worse HRQoL (β = -0.19, *p* < 0.001). However, this association was not moderated by perceived social support, benefits, or stress management ability.

#### Mediation hypotheses

In step 2, indirect effects through the proposed mediators were added to the model and evaluated (CFI = 0.87. TLI = 0.86. RMSEA = 0.04. SRMR = 0.05). As hypothesized, participants with a greater number of adverse COVID-19 experiences reported greater COVID-19 psychosocial and practical concerns on the mediating variables, including worse COVID-related anxiety (β = 0.15, *p* < 0.001), depression (β = 0.21, *p* < 0.001), health disruptions (β = 0.29, *p* < 0.001), daily disruptions (β = 0.25, *p* < 0.001), and financial burden (β = 0.33, *p* < 0.001). In turn, those who experienced worse COVID-related depression (β = − 0.49, *p* < 0.001) and financial burden (β = − 0.13, *p* = 0.003) had worse HRQoL. Moreover, an indirect effect through COVID-related depression (β = − 0.10, percentile bootstrap 95% CI − 0.15 to − 0.07) and financial burden (β = − 0.04, percentile bootstrap 95% CI − 0.07 to − 0.01) significantly accounted for the association between adverse COVD-19 experiences and worse HRQoL (total effect: β = − 0.19, *p* < 0.001; direct effect: β = − 0.02, *p* = 0.527).

#### Covariate findings

Several covariates had significant associations with the mediators and dependent variable (Table 3). Older age was associated with less COVID-related anxiety (β =− 0.19, *p* < 0.001), depression (β =− 0.31, *p* < 0.001), health disruptions (β =− 0.15, *p* < 0.001), daily disruptions (β =− 0.17, *p* < 0.001), and financial burden (β =− 0.27, *p* < 0.001), but worse HRQoL (β =− 0.07, *p* = 0.029). Similarly, being vaccinated against COVID-19 was associated with more COVID-related anxiety (β = 0.14, *p* < 0.001), depression (β = 0.14, *p* < 0.001), health disruptions (β = 0.07, *p* = 0.044), and daily disruptions (β = 0.18, *p* < 0.001), but better HRQoL (β = 0.10, *p* < 0.001). Compared to men, women had worse COVID-related anxiety (β = 0.09, *p* = 0.013), depression (β = 0.14, *p* < 0.001), and health disruptions (β = 0.10, *p* = 0.004), but also better HRQoL (β = 0.08, *p* = 0.005). Table 3 displays the standardized betas and *p*-values for each predictor across models.

**Table 3:** Regression models predicting HRQOL and proposed mediators

Model tested	Step 1		Step 2											
Predictor	HRQOL		HRQOL		Covid Anxiety		Covid Depression		Health Disruptions		Daily Disruptions		Financial Burden	
	β	*P*	β	*P*	β	*P*	β	*P*	β	*P*	β	*P*	β	*P*
Adverse Experiences	− **0.19**	** < .001**	− 0.02	.527	**0.15**	** < .001**	**0.21**	** < .001**	**0.29**	** < .001**	**0.25**	** < .001**	**0.33**	** < .001**
Age	**0.15**	** < .001**	− **0.07**	**.029**	− **0.19**	** < .001**	− **0.31**	** < .001**	− **0.15**	** < .001**	− **0.17**	** < .001**	− **0.27**	** < .001**
Female gender	0.03	.446	**0.08**	**. 005**	**0.09**	**.013**	**0.14**	** < .001**	**0.10**	**.004**	0.07	.099	− 0.05	.135
Diverse race or ethnicity	− 0.06	.067	− 0.05	.060	0.01	.645	− 0.01	.701	0.06	. 104	0.03	.543	0.07	.053
Years since dx	**0.07**	**.047**	**0.07**	**.009**	**0.07**	**.039**	0.02	. 482	0.03	.382	0.04	.395	0.00	.946
Vaccinated for covid	0.01	.657	**0.10**	** < .001**	**0.14**	** < .001**	**0.14**	** < .001**	**0.07**	**.044**	**0.18**	** < .001**	0.01	.960
Days since vaccine available	− 0.05	.158	− **0.06**	**.038**	0.01	.731	− 0.03	.433	− 0.02	.497	− 0.07	.105	0.02	.652
Perceived benefits	− 0.01	.899	0.04	.383										
Social support	0.04	.567	0.11	.145										
Stress management	**0.39**	** < .001**	**0.18**	**.008**										
AdvrsExp*benefit	− 0.01	.774	0.00	.939										
AdvrsExp*soc_supp	− 0.07	.292	− 0.06	. 391										
AdvrsExp*StressManage	0.09	.152	0.05	.439										
Covid anxiety			0.03	.614										
Covid depression			− **0.49**	** < .001**										
Health disruptions			0.03	.489										
Daily disruptions			− 0.16	.070										
Financial burden			**−0.13**	**.003**										

Adverse experiences total and indirect effects	β	*P*	Percentile bootstrap 95% CI
Total	− **0.19**	** < .001**	**[**− **0.25, −0.12]**
Indirect, COVID anxiety	0.00	.629	[− 0.01, 0.02]
Indirect, COVID depression	− **0.10**	** < .001**	**[−0.15, −0.07]**
Indirect, health disruptions	0.01	.493	[− 0.02, 0.04]
Indirect, daily disruptions	− 0.04	.094	[− 0.09, 0.00]
Indirect, financial burden	− **0.04**	**.004**	**[**− **0.07, **− **0.01]**

Bold text indicates statistically significant values

*N* = 1,043. *AdvrsExp* Adverse experiences index variable. *Soc_supp* Perceived social support. *β* Standardized regression coefficient. *CI* Confidence interval

## Discussion

This study found that adverse COVID-19 experiences were common in a sample of cancer survivors surveyed between June 2021 and March 2022. Moreover, those who reported a greater number of adverse COVID-19 experiences had lower HRQoL. Results of our model suggest that this may be due, at least in part, to an indirect effect through COVID-related depression and financial burden; those with more adverse COVID-19 experiences had more COVID-related depression and financial burden, and those with more depression and financial burden, in turn, had worse HRQoL. This suggests that clinicians should direct attention toward assessing patients' COVID-19 stressors and health systems need expanded resources for depression treatment and financial support.

### Main findings

Our results provide important descriptive data on how cancer survivors have experienced the COVID-19 pandemic. From a diverse list of 21 options, participants reported an average of more than three adverse COVID-19 experiences since the start of the pandemic. While the most common experience was having a friend, co-worker, or neighbor who tested positive for COVID-19 (73%), a significant number of participants also reported experiences that may have more serious implications. These included being at risk for severe COVID-19 outcomes due to older age (49%) or comorbidities (34%), having to delay a medical appointment (35%), experiencing decreased income due to the pandemic (22%), and having a friend, co-worker or neighbor die of COVID-19 (16%). These findings illustrate how many cancer survivors experienced increased stressors during the pandemic, putting them at heightened risk for poor physical, psychosocial, and financial outcomes.

This was one of the first known studies to conduct a comprehensive survey of cancer survivors' adverse COVID-19 experiences, pandemic-related psychosocial risk and resiliency factors, and the widely studied outcome of HRQoL. Our results reflect prior research showing that the stress of the COVID-19 pandemic can lead to more severe emotional distress and worse HRQoL in cancer survivors [6–9]. However, this study was unique in expanding its focus to include other plausible risk and resiliency factors, and simultaneously testing hypothesized associations in a comprehensive explanatory model using structural equation modeling. We found that a greater number of adverse COVID-19 experiences was associated with more severe scores on all psychosocial risk factors included in the survey. In turn, COVID-related depression and financial burden were significantly associated with worse HRQoL. This is consistent with a large body of research underscoring the role of depression in the health and well-being of cancer survivors [27, 28]. Recent studies have found that the pandemic has also contributed to financial burden in both mid- to high-income and low-income samples of cancer survivors [29, 30]. This could be a result of job loss, decreased income, or increased credit card debt directly attributed to the pandemic [29, 30]. In addition, the pandemic has had other negative effects that can indirectly lead to financial burden, such as healthcare disruptions leading to increased utilization of costly health services, caregiving demands leading to decreased productivity, or financial and emotional costs of bereavement [31]. Our findings build on accumulating evidence that the pandemic has exacerbated the financial toxicity of cancer care and that financial toxicity is associated with negative health outcomes in cancer survivors [27, 28, 32, 33].

There were other findings of the study that also warrant discussion, including several covariates in the model that were significant predictors of HRQoL or the proposed mediators. For example, being vaccinated against COVID-19 was associated with higher levels of COVID-related anxiety, depression, and disruptions. One plausible explanation based on prior research [34, 35] is that individuals who are more worried about COVID-19 are also more likely to take health precautions such as getting vaccinated as well as social distancing that can cause disruptions in their daily lives and healthcare experiences. Contrary to hypotheses, the psychosocial resiliency factors of social support, benefit-finding, and perceived ability to manage stress did not moderate the association between adverse COVID-19 experiences and HRQoL. Although past research prior to the onset of the pandemic suggested that these factors were often helpful for mitigating poor cancer outcomes [11, 12], it is possible that the COVID-19 pandemic was a new type of stressor that was not mitigated by traditional support systems. In that case, these protective factors may have no longer had a significant impact due to the unique challenges to coping with compound stressors posed by the COVID-19 pandemic. However, we cannot rule out the possibility that this was a Type II error, and we caution against drawing strong conclusions based on null results of a single study. Future work should seek to replicate our analyses, especially in larger and more representative samples.

### Strengths and limitations

This study had strengths and limitations. Key strengths included its relatively large sample size of individuals with a history of cancer and the use of a comprehensive assessment of COVID-19 experiences, practical concerns, and psychosocial risk and resiliency factors. In addition, we employed structural equation modeling to confirm the underlying measurement structure of a relatively new scale in our sample, and then to simultaneously test potential moderators and mediators of the pandemic's impact on HRQoL. The model employed Full Information Maximum Likelihood Estimation (FIML) and bootstrapping, powerful statistical methods to reduce bias when including variables with missing data and when estimating indirect effects.

However, several study limitations also warrant discussion. First, the study used a convenience sample from Northwestern Medicine and results may have been prone to sampling bias. The Northwestern Medicine patient population is already characterized by high socio-economic status compared to other regions in the U.S., and our specific sample had an even higher income and higher rates of college education, insurance coverage, and non-Latino White patients compared to the overall population from which they were recruited. Therefore, results cannot be generalized beyond adults in the U.S. with similar backgrounds to the present sample. Since the COVID-PPE measure was developed in a similar majority White sample of cancer survivors from large metropolitan areas [18], additional research needs to be conducted to examine if its psychometric properties and the findings in this paper generalize to other populations that are more diverse with respect to socioeconomic status, race, ethnicity, geography, and socio-political environment (e.g., differences in responses to the pandemic, different levels of public services devoted to healthcare and financial support). In addition, construct validity studies are still needed to compare the performance of the Adverse Experiences measure and the COVID-PPE scales to other measures of similar constructs. For example, future studies should replicate these analyses with more established measures of financial toxicity, such as the FACIT-COST [36], to further evaluate the generalizability of results and the construct validity of the financial burden measure used in the present study. Finally, the study employed a cross-sectional design, and follow-up studies with longitudinal data should be conducted to corroborate our hypothesized mediation model.

### Clinical implications

Our results have implications for informing supportive care programs in oncology so they can be responsive to ongoing COVID-related stressors experienced by cancer survivors. Future COVID-19 waves may disproportionately impact patients with cancer and other high-risk health conditions, due to potential disruptions in their healthcare and the potential for waning effectiveness of COVID-19 treatments intended for these populations (e.g., EvuSheld, monoclonal antibodies) against new subvariants of SARS-CoV-2 [37]. Such disruptions and medical vulnerabilities have the potential to decrease cancer survivors' HRQoL. Therefore, cancer clinics should continue to offer supportive services that are responsive to COVID-19 impact on their patients. For instance, clinics could query patients about their COVID-19-related experiences and stressors in order to allocate appropriate resources (e.g., telehealth services that decrease COVID-19 exposure risks for patients with medical vulnerabilities). Our study suggests that adverse experiences put patients at greater risk for depression and financial burden related to the pandemic, which can have downstream effects on HRQoL. Therefore, clinics may wish to devote resources towards screening for and treating depression, as well as financial navigation with services such as financial and legal counseling, transportation assistance, or telehealth.

In conclusion, this study found that greater adverse COVID-19 experiences were associated with higher depressive symptoms and financial concerns about COVID-19, and in turn, worse HRQoL. As the pandemic continues, oncology clinics should be cognizant of the experience of adverse COVID-19 events when allocating depression and financial support resources.

## Supplementary Information

**Additional file 1 Table S1. **Factor loadings.

## Data Availability

The datasets used and/or analysed during the current study are available from the corresponding author on reasonable request.
