# Supplementary material for: Adverse COVID-19 experiences and health-related quality of life in cancer survivors: indirect effects of COVID-19-related depression and financial burden
Source: J Patient Rep Outcomes. 2023 Jul 17;7:71. doi: 10.1186/s41687-023-00601-y (PMC10352476; doi:10.1186/s41687-023-00601-y)
Supplement: Supplementary file 1 — Additional file 1 Table S1. Factor loadings. [file 41687_2023_601_MOESM1_ESM.docx]

**Supplemental Table S1.** Factor Loadings

| **Domain, Item** | **Standardized Factor Loading** |
| --- | --- |
| COVID Anxiety |  |
| Covid-19 PPE Q1 | 0.837 |
| Covid-19 PPE Q2 | 0.635 |
| Covid-19 PPE Q3 | 0.746 |
| Covid-19 PPE Q4 | 0.785 |
| Covid-19 PPE Q5 | 0.594 |
| Covid-19 PPE Q6 | 0.593 |
| COVID Depression |  |
| Covid-19 PPE Q8 | 0.772 |
| Covid-19 PPE Q9 | 0.799 |
| Covid-19 PPE Q10 | 0.683 |
| Covid-19 PPE Q11 | 0.638 |
| Covid-19 PPE Q12 | 0.774 |
| Covid-19 PPE Q13 | 0.690 |
| COVID Health Disruption |  |
| Covid-19 PPE Q14 | 0.877 |
| Covid-19 PPE Q15 | 0.711 |
| COVID Daily Disruption |  |
| Covid-19 PPE Q18 | 0.611 |
| Covid-19 PPE Q19 | 0.546 |
| Covid-19 PPE Q20 | 0.665 |
| COVID Financial Burden |  |
| Covid-19 PPE Q24 | 0.834 |
| Covid-19 PPE Q25 | 0.562 |
| Covid-19 PPE Q26 | 0.756 |
| Covid-19 PPE Q27 | 0.767 |
| Perceived Benefits |  |
| Covid-19 PPE Q29 | 0.686 |
| Covid-19 PPE Q30 | 0.931 |
| Covid-19 PPE Q31 | 0.901 |
| Covid-19 PPE Q32 | 0.631 |
| Social Support |  |
| Covid-19 PPE Q35 | 0.662 |
| Covid-19 PPE Q36 | 0.423 |
| Covid-19 PPE Q37 | 0.538 |
| Covid-19 PPE Q38 | 0.475 |
| Stress Management |  |
| Covid-19 PPE Q39 | 0.418 |
| Covid-19 PPE Q40 | 0.665 |
| Covid-19 PPE Q41 | 0.580 |
| Covid-19 PPE Q42 | 0.782 |
| Covid-19 PPE Q42 |  |
| Health-related QoL |  |
| FACT-G7_1 (R) | 0.733 |
| FACT-G7_2 (R) | 0.590 |
| FACT-G7_3 (R) | 0.404 |
| FACT-G7_4 (R) | 0.540 |
| FACT-G7_5 | 0.538 |
| FACT-G7_6 | 0.724 |
| FACT-G7_7 | 0.718 |
